# Impact of simulation-based training on bougie-assisted cricothyrotomy technique: a quasi-experimental study

**DOI:** 10.1186/s12909-024-05285-6

**Published:** 2024-03-29

**Authors:** Ying Zhou, Huibin Gao, Qianyu Wang, Juan Zhi, Quanle Liu, Weipeng Xia, Qirui Duan, Dong Yang

**Affiliations:** Department of Anesthesiology, Plastic Surgery Hospital, Chinese Academy of Medical Sciences, Peking Union Medical College, Shijingshan District, Beijing, 100144 China

## Abstract

**Background:**

Cricothyrotomy is a lifesaving surgical technique in critical airway events. However, a large proportion of anesthesiologists have little experience with cricothyrotomy due to its low incidence. This study aimed to develop a multisensory, readily available training curriculum for learning cricothyrotomy and evaluate its training effectiveness.

**Methods:**

Seventy board-certificated anesthesiologists were recruited into the study. Participants first viewed an instructional video and observed an expert performing the bougie-assisted cricothyrotomy on a self-made simulator. They were tested before and after a one-hour practice on their cricothyrotomy skills and evaluated by a checklist and a global rating scale (GRS). Additionally, a questionnaire survey regarding participants’ confidence in performing cricothyrotomy was conducted during the training session.

**Results:**

The duration to complete cricothyrotomy was decreased from the pretest (median = 85.0 s, IQR = 72.5–103.0 s) to the posttest (median = 59.0 s, IQR = 49.0–69.0 s). Furthermore, the median checklist score was increased significantly from the pretest (median = 30.0, IQR = 27.0-33.5) to the posttest (median = 37.0, IQR = 35.5–39.0), as well as the GRS score (pretest median = 22.5, IQR = 18.0–25.0, posttest median = 32.0, IQR = 31.0-33.5). Participants’ confidence levels in performing cricothyrotomy also improved after the curriculum.

**Conclusion:**

The simulation-based training with a self-made simulator is effective for teaching anesthesiologists to perform cricothyrotomy.

## Introduction

Cricothyrotomy is a life-saving procedure to obtain access to the airway when routine methods (e.g., endotracheal intubation or laryngeal mask) are failed or contraindicated. In a “cannot intubate, cannot oxygenate” (CICO) scenario, it has been considered as a rapid and reliable method to establish the front-of-neck access for oxygenation restoration [1, 2]. The cricothyrotomy techniques involve incising the cricothyroid membrane and placing an artificial airway through the incision. They are generally categorised as surgical, percutaneous, and needle approaches [3]. Of note, although opinions about the best choice of performing cricothyrotomy under emergencies are conflicting [4, 5], there are several studies and published guidelines recommend the bougie-assisted surgical technique [6–9]. Additionally, according to an exploratory research using the “Airway APP” smartphone application for retrospective data collection, bougie-assisted cricothyrotomy was the most common choice when an emergency front-of-neck airway was needed [10].

According to the practice guidelines that were recently updated by the American Society of Anesthesiologists (ASA) [11], it is recommended to ensure that an invasive airway is performed by trained individuals whenever needed. Therefore, optimizing the training methods is crucial for maintaining procedural competence of cricothyrotomy among healthcare professionals. Since the incidence of cricothyrotomy in all intubations is only about 0.23% [12], access to clinical training is rare [13, 14]. Thus, training this skill is usually done with animal larynxes, manikins, or benchtop models [15–17]. However, a major disadvantage of these models is their short-term usability, which is attributed to tissue decay or destruction of anatomical structures caused by repeated practice. In this study, we designed a low-cost, readily available simulator and simulation-based training program, aiming to develop an effective project to promote anesthesia providers’ technical skills and confidence for cricothyrotomy.

## Materials and methods

### Study design

This was a single-arm, pretest-posttest design study with repeated measures based on time.

### Ethics

This study was conducted at Plastic Surgery Hospital, Peking Union Medical College. Since it does not meet the definition of clinical trial according to the International Committee of Medical Journal Editors, this study was not registered in any public trial registries but approved by the local institution’s ethics committee. Written consent was provided by each of the participants.

### Participants

Subjects were recruited from a one-day departmental CICO workshop held in June 2016 and July 2019, respectively. All of the participants were board-certified anesthesiologists. Before the training program, participants completed questionnaires to provide demographic, clinical experience information, and their experience with cricothyrotomy.

### Cricothyrotomy simulator

**Fig. 1:**
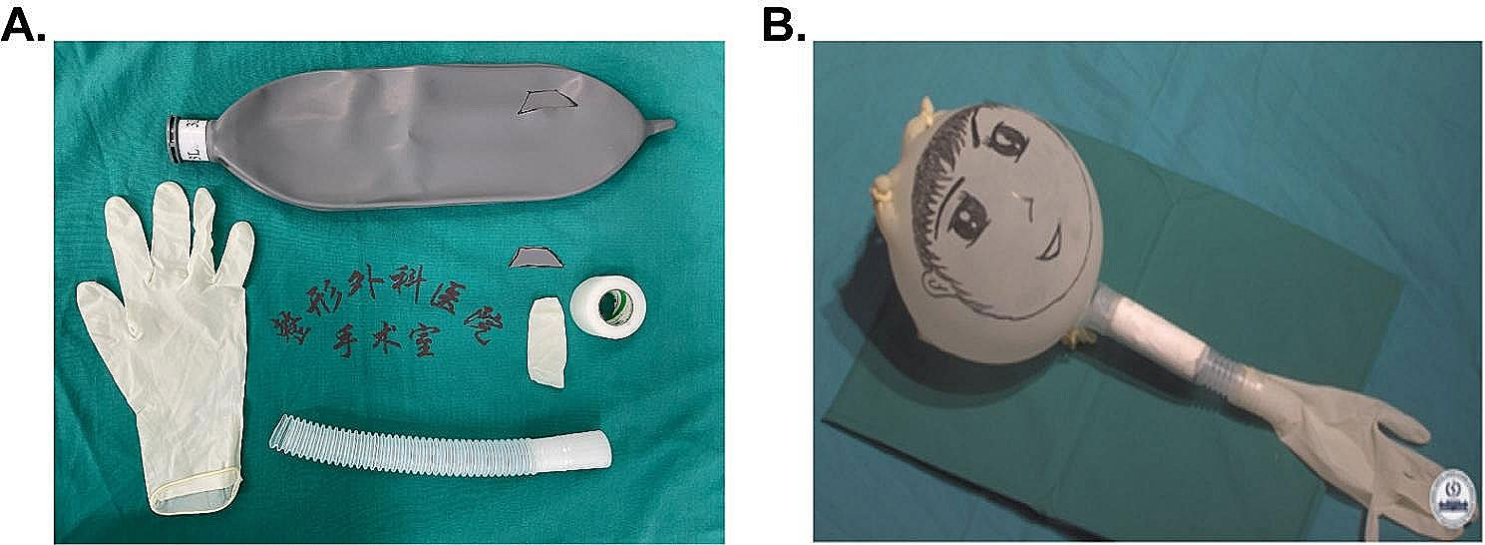
A self-made simulator is used for hands-on cricothyrotomy training section. (**A**) Materials needed to build the simulator. (**B**) Outlook of the finished model

The self-made cricothyrotomy simulator in the present study was made of readily available, low-cost materials. A piece of standard plastic breathing tube (20 cm long) was used to simulate the trachea. A trapezoid cut (1.5 cm wide on the upper side and 1 cm wide on the lower side; 1 cm high) on the breathing tube was to simulate the cricothyroid membrane space. A film cut from the disposable latex breathing bag was covered on the trapezoid cut and taped to the breathing tube, simulating the thick and strong cricothyroid membrane. Three to five layers of paper towel were used to represent connective tissue, and the finger cots of the latex glove were wrapped around the tube to simulate the skin (Fig. 1).

### Training protocol

**Fig. 2:**
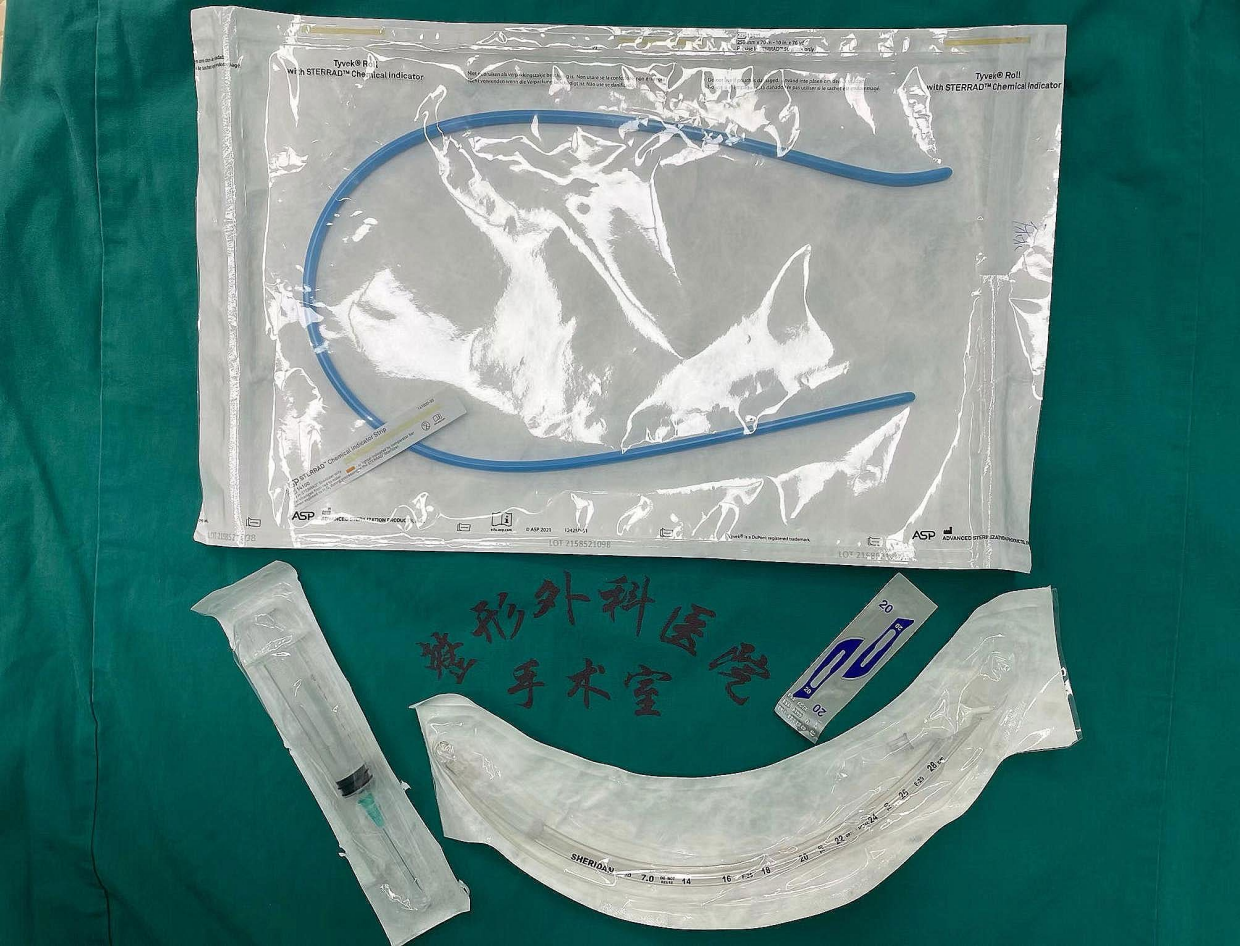
Equipment for the bougie-assisted scalpel technique

Considering that bougie-assisted cricothyrotomy is recommended by some guidelines [9, 18], and it is the technique we have the most experience with, we chose this technique for the training. At the beginning of the seminar, all participants were shown to a cricothyrotomy educational video produced by the authors. The video includes a three-dimension anatomy illustration of the cricothyroid membrane, an introduction and interpretation of Difficult Airway Society 2015 guidelines for the management of unanticipated difficult intubation in adults (2015 DAS guideline) [18], and a demonstration of how to perform surgical, percutaneous and needle cricothyrotomy on cadavers. Next, an instructor who was an expert in difficult airway management performed bougie-assisted cricothyrotomy on the self-made simulator. A size 10 scalpel, a bougie and a 6.5 mm cuffed endotracheal tube were prepared for the procedure (Fig. 2), and a “laryngeal handshake” to locate cricothyroid membrane with the rapid “stab, twist, bougie, tube” method was used here. During this process, the instructor detailed the indications and contraindications, preparation of equipment, technical skills, and complications. Participants were free to ask any questions to the instructor. Then, participants engaged in a timed pretest to perform bougie-assisted cricothyrotomy on the simulator. Their performance was evaluated by a skills checklist (Table 1) and a global rating scale (GRS) (Table 2). This was followed by an interactive practice session for 1 h, which allowed participants to practice repeatedly cricothyrotomy on the simulators with faculty feedback. Finally, participants took the posttest and scored with the same checklist and GRS.

**Table 1:** Checklist for bougie assisted cricothyrotomy performance

Task	Score		
	0: did not perform	1: inadequately perform	2: adequately perform
Direct assistants to ensure ventilation and oxygenation through the upper airway to the extent possible			
Prepare equipment			
Position the patient to lie supine and extend the neck as much as possible			
Identify relevant anatomy for cricothyrotomy			
Prepare neck with a skin cleaning agent			
Stabilize the larynx with the non-dominant hand			
Palpate thyroid cartilage and cricoid cartilage			
Make incision on the skin and subcutaneous tissues over the cricothyroid membrane with scalpel			
Make incision through the cricothyroid membrane			
Dilate the incision bluntly			
Insert the curved bougie via the incision			
Advance the bougie into the trachea			
Insert the endotracheal tube into the trachea along the bougie			
Confirm the proper location of endotracheal tube			
Remove the bougie			
Inflate the cuff of the endotracheal tube			
Resume ventilation using the airway			
Confirm the appropriate tube placement			
Secure endotracheal tube			
Reassess tube placement			

**Table 2:** Global rating scale

	Score				
	1	2	3	4	5
Preparation for procedure	Did not organize equipment well. Had to stop procedure frequently to prepare equipment.		Equipment generally organized. Occasionally had to stop and prepare items.		All equipment neatly organized, prepared, and ready for use.
Respect for tissue	Frequently used unnecessary force on tissue or caused damage.		Careful handling of tissue but occasionally caused inadvertent damage.		Consistently handled tissues appropriately with minimal damage.
Time and motion	Many unnecessary moves.		Efficient time/motion but some unnecessary moves.		Clear economy of movement and maximum efficiency.
Instrument handling	Repeatedly made tentative or awkward moves with instruments.		Competent use of instruments but occasionally appeared stiff or awkward.		Fluid moves with instruments and no awkwardness.
Flow of procedure	Frequently stopped procedure and seemed unsure of next move.		Demonstrated some forward planning with reasonable progression of procedure.		Obviously planned course of procedure with effortless flow from one move to the next.
Knowledge of procedure	Deficient knowledge.		Knew all important steps of procedure.		Demonstrated familiarity with all aspects of procedure.
Performance	Very poor.		Competent.		Clearly superior.

### Measurement

All the performance of participants in the pretest and posttest were video-recorded. To avoid bias, the camera’s field of view was restricted to technical procedures, and the participants’ faces were not filmed. Each video was viewed and assessed by two experts with experience in difficult airway management. The duration required to complete the cricothyrotomy, defined as the interval from identification of cricothyroid membrane to achievement of ventilation via the inserted endotracheal tube, was recorded in seconds. The technical skills were scored by a checklist (0 to 40) and a GRS (7 to 35) developed based on previous studies [19, 20]. Additionally, participants were asked to complete a 5-point Likert scale (from 1: very unconfident to 5: very confident) to assess their self-confidence in cricothyrotomy at three time points: before the seminar (baseline survey), before the pretest, and after the posttest.

### Statistics

Results were described as counts and frequencies (%) for qualitative data. Median and interquartile range (IQR) for quantitative data. The duration of cricothyrotomy, GRS score, checklist score and confidence level at each timepoint were evaluated with Friedman’s test, and Wilcoxon signed-rank test was used for post hoc analysis. Data analyses were performed with GraphPad 8.0 (GraphPad Software, Inc., San Diego, CA), and significance was set at *P* < 0.05.

## Results

A total of 74 anesthesiologists were recruited in the study, and 7 of them dropped out during the training. Finally, 67 subjects had finished the training procedure and all the tests. The demographic characteristics and experience performing cricothyrotomy of participants are shown in Table 3. There were 56.7% (*n* = 38) of the participants had less than 5 years of clinical experience, and 43.3% of them (*n* = 29) were more experienced physicians. However, most participants had no experience with real-life cricothyrotomy (*n* = 60, 89.6%), and only 28.4% of them (*n* = 48) had ever received training regarding this skill (*n* = 48, 71.6%).

**Table 3:** Sample characteristics and clinical experience of cricothyrotomy (*N* = 67)

Sample characteristics (*N* = 67)		n (%)
Gender	Female	37 (55.2)
	Male	30 (44.8)
Experience in anesthesia (years)	< 5	38 (56.7)
	5–10	20 (29.9)
	> 10	9 (13.4)
Numbers of cricothyrotomies performed in patients	0	60 (89.6)
	1	5 (7.5)
	2–4	0 (0)
	≥ 5	2 (2.9)
Numbers of previous training on cricothyrotomy in the past 10 years	Never	48 (71.6)
	Once	15 (22.4)
	2–4 times	2 (3.0)
	≥ 5 times	2 (3.0)

**Fig. 3:**
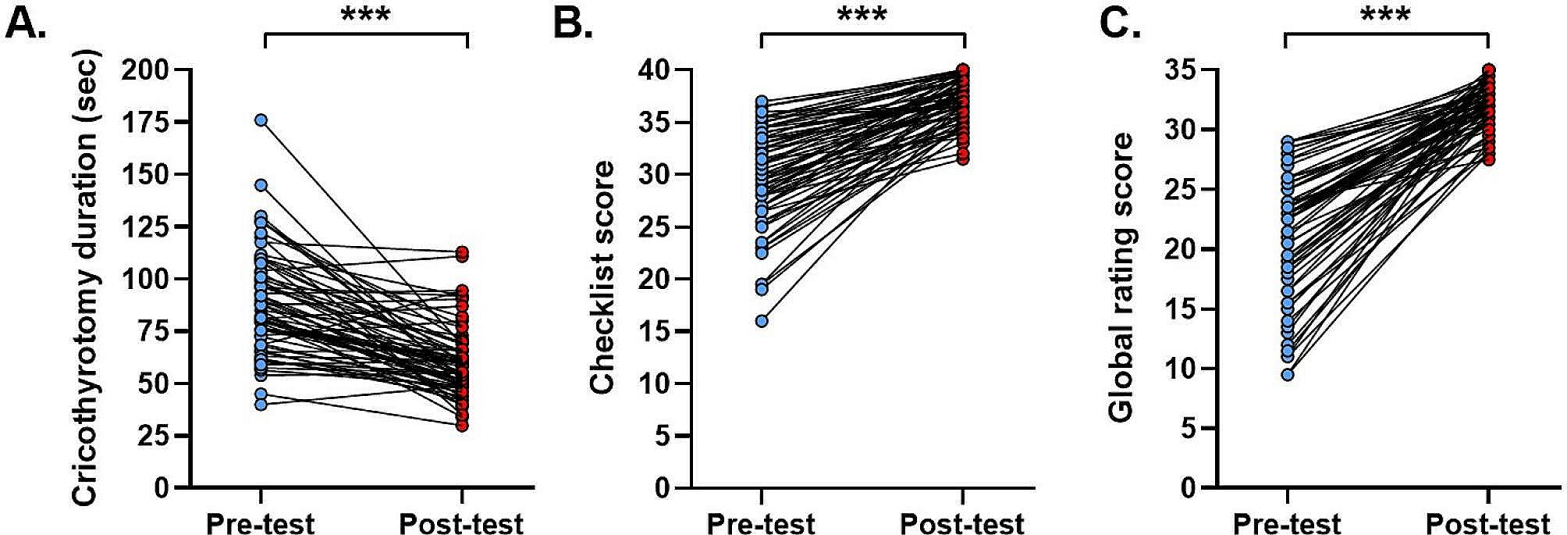
Duration to perform cricothyrotomy (**A**), scores of the GRS (**B**) and checklist (**C**) at pretest and posttest. Each dot represents an individual

**Fig. 4:**
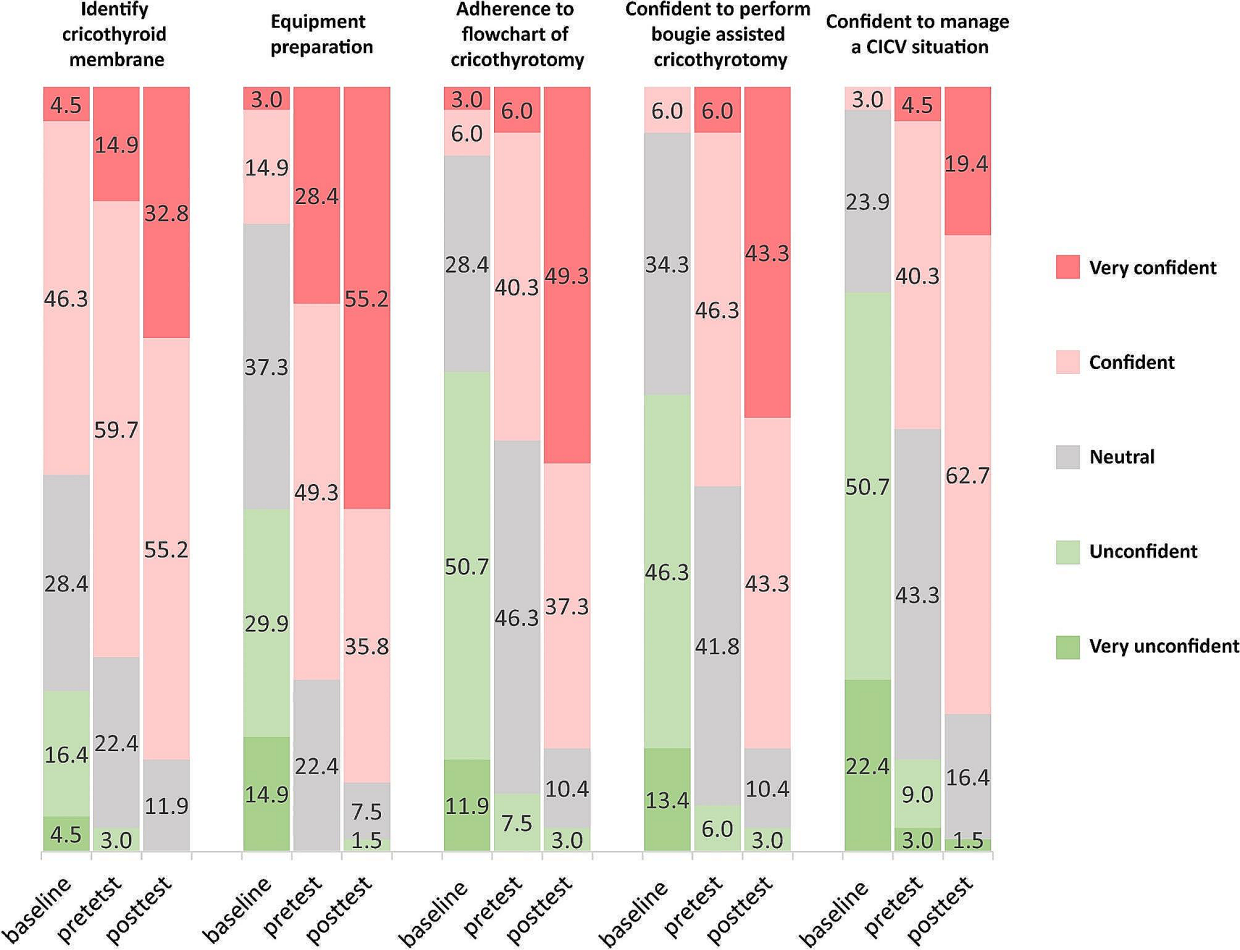
Confidence level of participants at the baseline, pretest and posttest

All the participants were able to accomplish the procedure, and the duration to complete cricothyrotomy was found to decrease from the pretest (median = 85.0 s, IQR = 72.5–103.0 s) to the posttest (median = 59.0 s, IQR = 49.0–69.0 s) (Fig. 3A). Furthermore, based on the previous studies, we combined a task-specific checklist and a GRS tool to assess the performance in bougie-assisted cricothyrotomy skills. There was a significant improvement in the median checklist score from the pretest (median = 30.0, IQR = 27.0-33.5) to the posttest (median = 37.0, IQR = 35.5–39.0) (Fig. 3B). The median GRS score also improved significantly from the pretest (median = 22.5, IQR = 18.0–25.0) to the posttest (median = 32.0, IQR = 31.0-33.5) (Fig. 3C). Moreover, results of the online surveys showed that after the theoretical learning, the level of self-confidence in identifying cricothyroid membrane, preparing equipment, complying with the flowchart of cricothyrotomy, performing cricothyrotomy and managing a CICO situation all improved significantly, and it increased further after practice session (Fig. 4). The most impressive gain was that only 3.0% of the participants (*n* = 2) were confident about managing a CICO scenario before the seminar, and it was increased to 82.1% (cumulative percentage of participants indicated they were “confident” or “very confident”, *n* = 55) after the seminar.

## Discussion

Our results emphasize the value of the simulation-based training program for the acquisition of procedural skills and confidence in bougie-assisted cricothyrotomy. After a one-day curriculum, the total time to perform cricothyrotomy was shortened, showing a trend of achieving within 40 s (the optimal time to perform cricothyrotomy proficiently [21]). Furthermore, the technical quality of the procedure, as assessed by the checklist score and the GRS, has improved significantly after the training. Despite a self-made, low-fidelity simulator used in our study’s practice session, the participants’ confidence to perform cricothyrotomy in real life and to manage a CICO situation increased significantly after the seminar.

CICO is a life-threatening emergency that requires rapid and decisive management. In such a crisis, cricothyrotomy can lead to effective ventilation and provide the maximal potential to resuscitate a patient. However, physicians usually have little experience performing one. Specifically, in China, more than 94% of anesthesiologists had no experience with cricothyrotomy, and only 13.6%${\sim}$24.9% of them had ever received relevant training [14]. Lack of skill maintenance opportunities results in skill degradation and poor confidence, which may lead to an unacceptable delay in the realisation of performing cricothyrotomy. Thus, regular training is recommended to be reinforced for every physician [18].

Simulation-based training is an effective and safe method to acquire skills and maintain professional competence in healthcare [22]. Evidence showed that simulation-based airway management training was superior in improving skill performance than non-simulation interventions, including lecture, video, clinical observation and so on [23]. Cadavers, animals or their larynxes have unique advantages as simulation resources because of their similar anatomical landmarks and texture to living patients [24–28]. However, the use of cadaveric and animal models is limited due to ethical issues or available access. Commercial manikins or small benchtop models are also high-fidelity simulators [29–31], but their high price and maintenance costs hinder their widespread. Interestingly, recent studies have utilized novel technologies, such as 3D printing and virtual reality, to develop task trainers for cricothyrotomy training [32, 33]. Despite the high fidelity and relatively low cost, these advanced simulators, which require specialized equipment and technicians, have not been popularized yet. Considering that a training task should be easy to implement in every hospital [34], we constructed a cricothyrotomy simulator with accessible materials in the operation rooms (including plastic breathing tubes, latex breathing bags, paper towels and latex gloves). The ready availability of these materials ensures that learners can repeat their practice to acquire and retain the cricothyrotomy skills easily. Participants’ performance, as measured by the checklist and GRS, was improved significantly after our curriculum, which is consistent with previous studies that also utilized similar self-made simulation models [35, 36]. These results suggest that our simulators achieved the aim of improving participants’ procedural skills, supporting the notion that a low-fidelity simulator is an effective tool in cricothyrotomy training.

The confidence of a physician in his or her cricothyrotomy skills is considered as a decisive factor in performing the procedure timely in a CICO event [18, 37]. Thus, we believe participants’ confidence level is another important outcome and assessed it through questionnaires. After finishing the training course, most participants reported positive effects on their confidence in cricothyrotomy. Notably, participants were more confident in performing this technique in real life, even though they practiced on a relatively simple simulator. The effect on improving self-confidence was similar to those training sessions using high-fidelity simulators [38–40]. This may be due to the adequate instruction throughout the training process. According to the analysis of previous research, the knowledge of cricothyrotomy did not seem to be effectively transferred to participants in the simulation curriculums [23, 41]. Therefore, we integrated non-simulation based training, including instructional videos and lectures, into our study. We also set the hands-on practice section as an instructor-led training in our curriculum, hoping to provide a more efficient training method in this field. In conclusion, these results highlighted the quality of our curriculum.

A major limitation of our study is that we did not examine skill retention after the training. This is because the optimal interval to examine the maintenance of cricothyrotomy skills is unknown. Sankaranarayanan et al. have reported that an interval of two weeks is a suitable washout time [42], but another study conducted the retention tests at 3, 6 and 12 months after training [43]. Further research should be performed to determine the best opportunity for examining skill retention, as well as the learning curve of cricothyrotomy. Another limitation is that we only focused on teaching procedural skills but neglected to train participants under relevant clinical scenarios. As a genuine CICO event is a time-critical task requiring immediate evaluation, decision and feedback, rehearsal and prospective assignment of respective responsibilities of the entire team members are recommended to address this issue [18]. Further curricula should include real-life CICO scenarios (such as CICO patients with morbid obesity or large neck haematomas caused by trauma) and multidisciplinary training.

In conclusion, we have shown that a simple simulation-based curriculum consisting of instructional video, lecture and hands-on practice can improve the performance and confidence of anesthesiologists in cricothyrotomy. Since the self-made simulators we utilized in our study can be easily reproduced, we hope that similar programs will be built in more and more hospitals for regular specific training for cricothyrotomy.

## Data Availability

All data generated or analysed during this study are included in this published article.
